# Fast Estimation of Defect Profiles from the Magnetic Flux Leakage Signal Based on a Multi-Power Affine Projection Algorithm

**DOI:** 10.3390/s140916454

**Published:** 2014-09-04

**Authors:** Wenhua Han, Xiaohui Shen, Jun Xu, Ping Wang, Guiyun Tian, Zhengyang Wu

**Affiliations:** 1 College of Automation Engineering, Shanghai University of Electric Power, Shanghai 200090, China; E-Mails: yuko1228@163.com (X.S.); daniel_xiaoxu@163.com (J.X.); zenkobe24@163.com (Z.W.); 2 College of Automation Engineering, Nanjing University of Aeronautics and Astronautics, Nanjing 210016, China; E-Mail: zeit@263.net; 3 School of Electrical and Electronic Engineering, Newcastle University, Newcastle upon Tyne, NE1 7RU, UK; E-Mail: g.y.tian@ncl.ac.uk

**Keywords:** nondestructive testing, magnetic flux leakage, affine projection, system identification

## Abstract

Magnetic flux leakage (MFL) inspection is one of the most important and sensitive nondestructive testing approaches. For online MFL inspection of a long-range railway track or oil pipeline, a fast and effective defect profile estimating method based on a multi-power affine projection algorithm (MAPA) is proposed, where the depth of a sampling point is related with not only the MFL signals before it, but also the ones after it, and all of the sampling points related to one point appear as serials or multi-power. Defect profile estimation has two steps: regulating a weight vector in an MAPA filter and estimating a defect profile with the MAPA filter. Both simulation and experimental data are used to test the performance of the proposed method. The results demonstrate that the proposed method exhibits high speed while maintaining the estimated profiles clearly close to the desired ones in a noisy environment, thereby meeting the demand of accurate online inspection.

## 1. Introduction

After the long-time operation of railway tracks or oil pipelines, many sorts of defects, like mechanical damage or cracks, may occur on their surfaces, which may trigger terrible accidents and lead to great financial losses and even personal casualties. To eliminate the hidden risks as much as possible, magnetic flux leakage (MFL) inspection, one of the most important and sensitive methods for electromagnetic nondestructive testing (NDT) of surfaces and near-surface area in ferromagnetic materials, can be widely used to detect such defects and is fully applied to make regular inspections [1–4].

MFL signals are acquired by an array of Hall effect sensors closely distributed above the surface of a measured object when the object is magnetically saturated by strong permanent magnets. For some facilities, such as an oil pipeline and railway track, inspections may be conducted at a large scale or a long distance. After removing noise from the acquired MFL signals by some signal processing methods, e.g., a modified wavelet transform domain adaptive FIR filtering algorithm [5], it is essential to find a fast and efficient feature extracting method to deal with the massive MFL signals. An online MFL inspection system consists of a high-speed MFL signal acquisition system and a fast feature extracting method. The former can be achieved by some technical means, e.g., a bobbin-type magnetic camera to inspect stress corrosion on a small-bore piping system at high speed [6] and an MFL detector with an advanced data processing system for long-range pipeline inspection [7].

The inverse method, a common method for MFL feature extraction, including a forward model and iterative algorithm, is of great interest. Artificial neural networks, the dipole model and the finite element model all have been used as a forward model [8–10]. Iterative algorithms include the gradient descent algorithm, the particle swarm optimization algorithm and the genetic algorithm [11–13]. Despite their favourable performance, they cannot reach the requirement of a short estimating time for online inspection. A fast inverse method used for estimating rectangular crack sizes has a computational cost of more than two seconds [12], and another fast method for arbitrary 2D defect profile reconstruction ranges from 14 to 64 s [1]. These inverse methods fail to extract features from MFL signals with high speed.

To shorten the time, rapid inspection methods are in great demand. Adaptive filtering is an important technique in many applications, such as system identification, channel equalization, echo cancellation and active noise control [14–16]. The well-known least mean squares (LMS) algorithm [17,18] was widely used for adaptive filtering, because of its robustness and efficiency. However, its obvious drawback is the over dependence on the statistics of their input signals. The affine projection algorithm (APA) [19] generalizes a normalized LMS adaptive filtering algorithm and can overcome the problem of LMS. In recent years, APAs have been suggested for adaptive system applications as an efficient alternative because of the fast convergence rate [20–22]. This work modified the standard APA to form a new filtering algorithm, named the multi-power APA (MAPA), for fast estimating defect profiles from MFL signals. It can identify precisely the relationship between the inputs (MFL signals) and the outputs (defect profile) after being trained by a certain amount of data, thus obtaining a result close to the true defect profiles. The proposed method, which advances significantly in the aspect of estimation speed, can totally meet the demands of the fast inspection of long-distance devices or facilities.

The presentation of this paper proceeds as follows: in Section 2, two kinds of extracted features of defects and their identification by APA are discussed. Section 3 presents the detailed process of MAPA and fast defect profile estimation from MFL signals based on MAPA. The simulations and experimental results are shown in Section 4. Finally, conclusions are given in Section 5.

## 2. Feature Extraction of Defects and System Identification by APA

**Figure 1.**
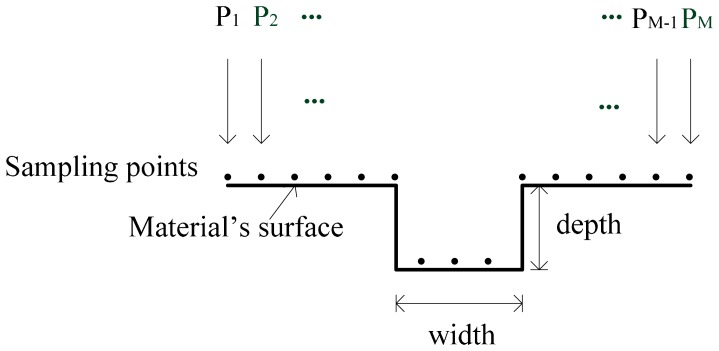
Two types of defect features (2D defect).

The extracting features of defects commonly consist of two types, defect size and defect profile, shown in Figure 1. Many studies [12,23] regard defects on the surface of materials as rectangles or slopes with a certain angle. Thus, the extracted features are just their length, width and depth, as well as angle, if needed. In this case, by regulating the parameters of a feature extracting model and the sizes of their paired defects, the trained model can extract the defect size information from MFL signals. This size extraction method is rather simple and easy to realize. Unfortunately, the shape of a surface's defect can be arbitrary in the real world. We can obtain the information of an arbitrary defect only when the defect profile consists of enough sampling points distributing on the surface.

To estimate the whole profile of a defect, processing every sampling point is inevitable. Thus, the available time to every point should be short enough to achieve the fast estimation of a defect profile.

**Figure 2.**
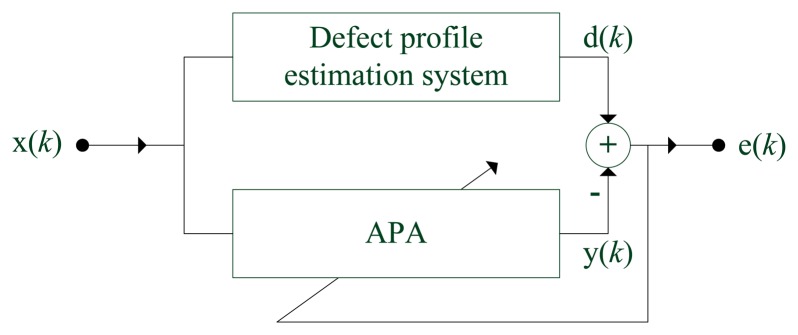
The system structure for identifying a defect profile by an affine projection algorithm (APA) filter.

System identification is the most common application of APA filters. The structure of a defect profile estimation system by APA is shown in Figure 2. Input *x(k)* is the MFL signal at the *k*-th sampling point. *d(k)* and *y(k)* are the desired depth and the output value of an APA filter, respectively. It is essential to regulate an APA weight vector in order to minimize the error between *d(k)* and *y(k)*. When error *e(k)* is small enough after several generations, the APA filter can replace the actual defect profile system to estimate the corresponding defect profile from MFL signals.

## 3. Multi-Power APA and Fast Estimation of Defect Profiles

In the defect profile estimation problem, different from time series or time-varying problems, the depth of the *k*-th sampling point is related with not only the MFL signals before the *k*-th sampling point, but also the ones after it. Conversely, we can conduct the analysis from the aspect of a defect leading to the significant change of MFL signals; the profile depth of the *k*-th sampling point and its former and latter ones decide the magnetic flux density of the *k*-th point together.

In addition, for an *N*-order adaptive filter, the *k*-th output is calculated by:

(1) $$y(k)=\sum_{i=0}^{N}w_{i}(k)x(k-i)={\text{w}}^{T}(k){\text{x}}_{ap}(k)$$

where **x***_ap_*(*k*) = [*x*(*k*) *x*(*k* − 1) … *x* (*k* − *N*)]*^T^* and **w**(*k*) = [*w*_0_(*k*) *w*_1_(*k*) … *w_N_*(*k*)]*^T^* are the *k*-th input vector and weight vector, respectively.

The *k*-th output of an adaptive filter is the sum of products between weight *w_i_(k)* and the first power of input *x(k−i)*, but for the defect profile estimation, the MFL density of the *k*-th sampling point is also influenced by the higher power of an input, e.g., *x^2^(k)* and *x^3^(k)*. To solve the problem of defect profile estimation, the corresponding improvements in view of the above two problems should be applied to APA.

### 3.1. Multi-Power APA (MAPA)

In some cases, reusing the past signals contributes to the fast convergence rate of an algorithm. Data reuse is an approach to speed up the convergence of an adaptive filtering algorithm if its inputs are correlative, according to [19].

In MAPA, we define the order of a MAPA filter as the sum of *N_1_* and *N_2_*, where *N_1_* means the use of the past *N_1_* inputs and *N_2_* means the use of *N_2_* inputs after the current one. The *N_1_* + *N_2_* inputs are all correlative with the current one. As *N_2_* inputs are added to figure out the current output, compared with the basic APA, the reuse number of inputs is also increased, not only the past *L_1_* inputs in the basic APA, but also *L_2_* inputs. According to the above description, the input matrix is given as follows:

(2) $$\begin{array}{l} \text{X}(k)=\left[\begin{array}{ccccccc} x(k+N_{2}+L_{2}) & \cdots & x(k+N_{2}+1) & x(k+N_{2}) & x(k+N_{2}-1) & \cdots & x(k+N_{2}-L_{1})\\ \vdots & \ddots & \vdots & \vdots & \vdots & ⋰ & \vdots \\ x(k+L_{2}+1) & \cdots & x(k+2) & x(k+1) & x(k) & \cdots & x(k-L_{1}+1)\\ x(k+L_{2}) & \cdots & x(k+1) & x(k) & x(k-1) & \cdots & x(k-L_{1})\\ x(k+L_{2}-1) & \cdots & x(k) & x(k-1) & x(k-2) & \cdots & x(k-L_{1}-1)\\ \vdots & ⋰ & \vdots & \vdots & \vdots & \ddots & \vdots \\ x(k-N_{1}+L_{2}) & \cdots & x(k-N_{1}+1) & x(k-N_{1}) & x(k-N_{1}-1) & \cdots & x(k-L_{1}-N_{1}) \end{array}\right]\\ =\left[\begin{array}{ccccccc} \text{x}(k+L_{2}) & \cdots & \text{x}(k+1) & \text{x}(k) & \text{x}(k-1) & \cdots & \text{x}(k-L_{1}) \end{array}\right] \end{array}$$

Considering that all of the inputs to the *P*-th power and the ones below the *P*-th power are related with the current output, the elements of input matrix **X**(*k*) should be further expanded. Specifically, *x(k)* is replaced by [*x*(*k*) *x*^2^(*k*) ⋯ *x^P^*(*k*)]*^T^*. After the transformation, **X**(*k*) is changed to be a *P*(*N_1_ + N_2_ + 1)*-by- (*L_1_ + L_2_ + 1)* matrix *X_mp_*(*k*):

(3) $$\begin{array}{l} {\text{X}}_{mp}(k)=\left[\begin{array}{ccccccc} \text{a}(k+N_{2}+L_{2}) & \cdots & \text{a}(k+N_{2}+1) & \text{a}(k+N_{2}) & \text{a}(k+N_{2}-1) & \cdots & \text{a}(k+N_{2}-L_{1})\\ \vdots & \ddots & \vdots & \vdots & \vdots & ⋰ & \vdots \\ \text{a}(k+L_{2}+1) & \cdots & \text{a}(k+2) & \text{a}(k+1) & \text{a}(k) & \cdots & \text{a}k-L_{1}+1)\\ x(k+L_{2}) & \cdots & x(k+1) & \text{a}(k) & \text{a}(k-1) & \cdots & \text{a}(k-L_{1})\\ \text{a}(k+L_{2}-1) & \cdots & \text{a}(k) & \text{a}(k-1) & \text{a}(k-2) & \cdots & \text{a}(k-L_{1}-1)\\ \vdots & ⋰ & \vdots & \vdots & \vdots & \ddots & \vdots \\ \text{a}(k-N_{1}+L_{2}) & \cdots & \text{a}(k-N_{1}+1) & \text{a}(k-N_{1}) & \text{a}(k-N_{1}-1) & \cdots & \text{a}(k-L_{1}-N_{1}) \end{array}\right]\\ =\left[\begin{array}{ccccccc} \text{A}(k+L_{2}) & \cdots & \text{A}(k+1) & \text{A}(k) & \text{A}(k-1) & \cdots & \text{A}(k-L_{1}) \end{array}\right] \end{array}$$

where **a**(*k*) = [*x*(*k*) *x*^2^(*k*) ⋯ *x^P^*(*k*)]*^T^*.

Then, the MAPA filter computes output *y_mp_(k)* as:

(4) $$\begin{array}{ll} {\text{y}}_{mp}(k) & ={\text{X}}_{mp}^{T}(k){\text{w}}_{mp}(k)\\ & ={\left[\begin{array}{ccccccc} y_{-L_{2}}(k) & \cdots & y_{-1}(k) & y_{0}(k) & y_{1}(k) & \cdots & y_{L_{1}}(k) \end{array}\right]}^{T} \end{array}$$

where **w***_mp_(k)* is a *P*(*N_1_ + N_2_ + 1)*-by-1 weight vector of the MAPA filter.

The error vector between *y_mp_(k)* and the desired output profile is as follows:

(5) $${\text{e}}_{mp}(k)=\text{d}(k)-{\text{y}}_{mp}(k)$$

where **d**(*k*) = [*d*(*k* + *L*_2_) ⋯ *d*(*k* + 1) *d*(*k*) *d*(*k* − 1) ⋯ *d*(*k* − *L*_1_)].

The aim of the MAPA filter is to minimize the error between **w***_mp_(k)* and **w***_mp_(k+1)* or a constrained optimization problem:

(6) $$\min\frac{1}{2}{\left\|{\text{w}}_{mp}(k+1)-{\text{w}}_{mp}(k)\right\|}^{2}$$

where the constraint condition is $\text{d}(k)-{\text{X}}_{mp}^{T}(k){\text{w}}_{mp}(k+1)=0$.

By applying the method of Lagrangian multipliers, the above problem can be transferred to an unconstrained problem.

(7) $$F({\text{w}}_{mp}(k+1))=\frac{1}{2}{\left\|{\text{w}}_{mp}(k+1)-{\text{w}}_{mp}(k)\right\|}^{2}+\lambda_{mp}^{T}(k)(\text{d}(k)-{\text{X}}_{mp}^{T}(k){\text{w}}_{mp}(k+1))$$

where $\lambda_{mp}^{T}(k)$ is an (*L_1_ + L_2_ + 1)*-by-1 vector of Lagrange multipliers at the *k*-th sampling point.

Then, as the gradient of *F(***w***_mp_(k+1))* to **w***_mp_(k+1)* is zero, the relationship between **w***_mp_(k+1)* and **w***_mp_(k)* is obtained:

(8) $${\text{w}}_{mp}(k+1)={\text{w}}_{mp}(k)+{\text{X}}_{mp}(k)\lambda_{mp}(k)$$

Deriving from Equation (5) and the constrained condition, we have:

(9) $${\text{w}}_{mp}(k+1)={\text{w}}_{mp}(k)+{\text{X}}_{mp}(k){\left({\text{X}}_{mp}^{T}(k){\text{X}}_{mp}(k)\right)}^{-1}{\text{e}}_{mp}(k)$$

In order to keep the balance between convergence rate and steady-state estimation error to improve the performance of APA, the step size is added [14]. For the simplicity and fast execution speed of MAPA, we define a fixed step size *μ*, and then Equation (8) is changed to:

(10) $${\text{w}}_{mp}(k+1)={\text{w}}_{mp}(k)+\mu {\text{X}}_{mp}(k){\left({\text{X}}_{mp}^{T}(k){\text{X}}_{mp}(k)\right)}^{-1}{\text{e}}_{mp}(k)$$

After the regulation of weights, the MAPA filter can replace the unknown system to obtain precise outputs close to the desired ones, *i.e.*, the true profile of a defect.

### 3.2. Fast Defect Profile Estimation from MFL Signal Based on MAPA

**Figure 3.**
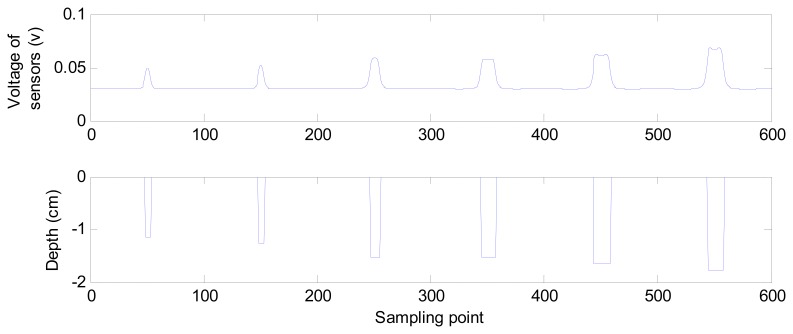
The depth and voltages acquired by sensors of 600 sampling points.

With the sensors scanning the surface of materials and sampling the magnetic flux density, the magnetic flux density at different sampling points is acquired and described as different voltages. Figure 3 shows the depth and voltages acquired at 600 consecutive sampling points. As the surface depth changes, different voltages are acquired by the sensors. We can say that a different magnetic flux density distributes on the surface.

To estimate the depth of a surface point-by-point or defect profiles by using MAPA, the voltages acquired by sensors at sampling points are regarded as the inputs of the MAPA filter, and the true depth at sampling points are treated as desired outputs. The first part of this method is to finish the regulation of the weight vector in the MAPA filter with the following steps:

Step 1, initialization of an MAPA filter: weight vector **w***_mp_*(0) is set as 0 and the step size is chosen in the range from zero to two. After the input and output series are given, the number of sampling points is determined as *M*. In addition, a weak disturbance is added by using a small constant *γ* to prevent the divisibility by zero and to ensure stability.
Step 2, calculation of an error vector: According to Equation (3), the *k*-th output vector of the MAPA filter is calculated. After that, use Equation (4) to obtain error vector **e***_mp_(k)* between the output vector and desired output profile vector.
Step 3, updating the weight vector: add the weak disturbance in the process of updating **w***_mp_(k)* to **w***_mp_(k+1)*:
  (11) $${\text{w}}_{mp}(k+1)={\text{w}}_{mp}(k)+\mu {\text{X}}_{mp}(k){\left({\text{X}}_{mp}^{T}(k){\text{X}}_{mp}(k)+\gamma \text{I}\right)}^{-1}{\text{e}}_{mp}(k)$$
  where **I** is the identity matrix of size *L_1_ + L_2_ + 1* and *γ*is an adjustment parameter.
Step 4, check the termination condition: if *k* exceeds the number of sampling points *M*, the result of the last generation **w***_mp_(M)* is the final weight vector, and otherwise, return to Step 2 for calculating the next sampling point *k + 1*.

Now, we can estimate a defect profile **R** with the MFL signals at *z* sampling points.

(12) $$\begin{array}{ll} \text{R} & ={\left[\begin{array}{cccc} \text{A}(1) & \text{A}(2) & \cdots & \text{A}(z) \end{array}\right]}^{T}{\text{w}}_{mp}(M)\\ & ={\left[\begin{array}{cccc} r_{1} & r_{2} & \cdots & r_{z} \end{array}\right]}^{T} \end{array}$$

## 4. Simulation and Experiment

The data used to verify the effect of the defect profile estimating method based on MAPA includes two parts: the simulation data generated by the software ANSYS and the data acquired by an experimental setup. The majority of the first part is used to finish the regulation of a weight vector in the MAPA filter, and then, both the remaining of the first part and the second part are applied to estimate defect profiles.

An Intel Core i7 2.20GHz laptop on a Win7 professional operating system is used. The parameters described in Section 3 are crucial for the performance of an MAPA filter. After several tests, the suitable values for the defect profile estimation are listed in Table 1.

### 4.1. Simulation Data and Results

**Figure 4.**
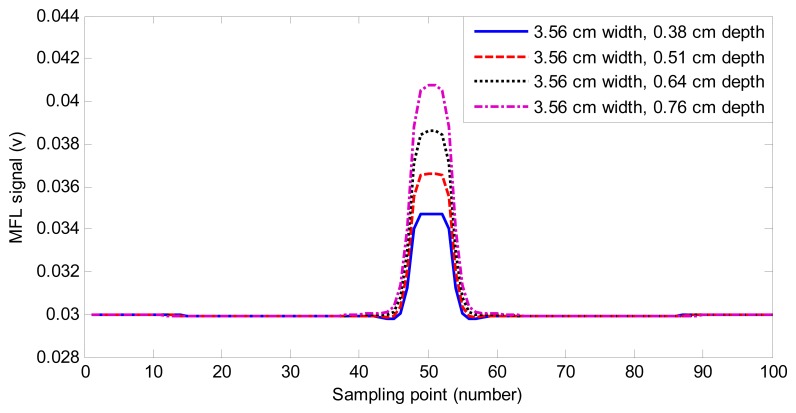
The Magnetic flux leakage (MFL) signals of four defects.

We generate the simulation data with the software ANSYS. The 2D MFL data includes 240 defect samples with varying widths and depths. Figure 4 shows the MFL signals of four defects. As can be found in Figure 4, MFL signals are different because of the change of the depth and width of defects. The information of defect profiles is clearly contained in MFL signals. Two-hundred thirty samples are used to regulate the weight vector of the MAPA filter, and the remaining 10 samples are used to estimate defect profiles. The size of the sampling point interval is 0.508 cm.

**Figure 5.**
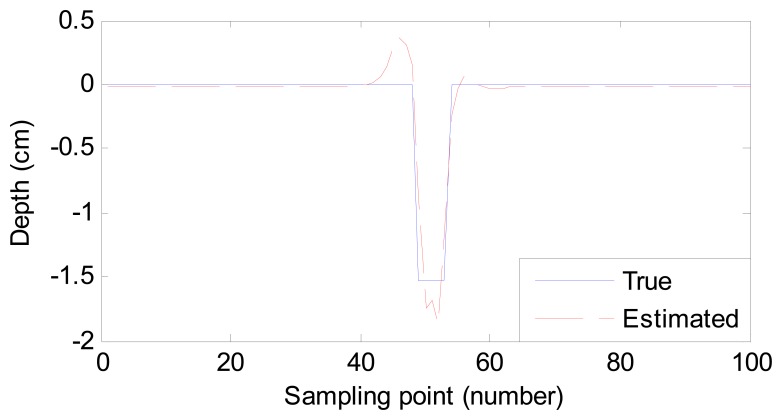
Comparison of the true profile and estimated profile (sample No. 2).

**Figure 6.**
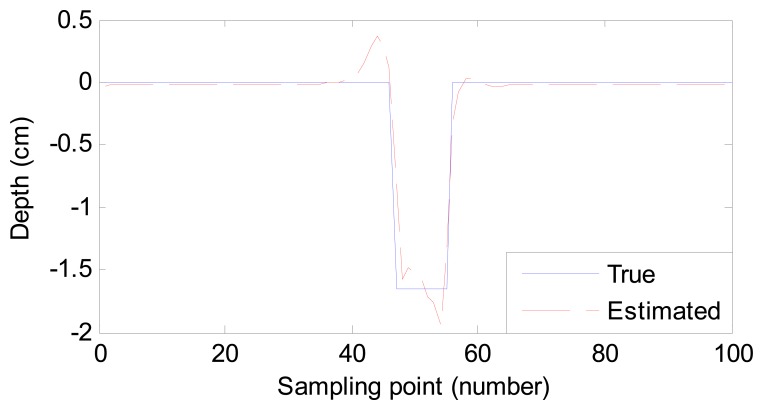
Comparison of the true profile and estimated profile (sample No. 6).

For a good description of the estimation results, we use root-mean-square error (RMSE) to quantize the difference between the estimated and desired profiles. The RMSE values of 10 samples and their estimation time are listed in Table 2. In addition, the process of adjusting the weight vector costs 1.098 s. Two of the estimated ten profiles for sample No. 2 and sample No. 6 are also shown in Figures 5 and 6, respectively.

From Table 2, Figures 5 and 6, we conclude that:

(1) Compared with the listed time in [1,12], the proposed method costs less time. Though the process of weight vector regulation costs 1.098 s, it needs to operate once only. Therefore, the estimating time meets the demand of fast estimation or even online inspection.
(2) Despite the size difference among defects, the RMSE values are small enough. In other words, the estimated defect profiles are close to the true ones. The robustness of the proposed method is high to estimate different defects.

### 4.2. Experimental Setup and Results

**Figure 7.**
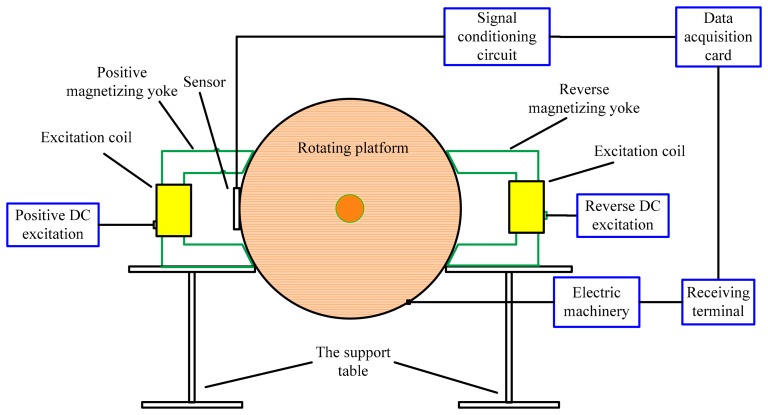
Schematic of the experimental equipment.

In order to further verify the effect of the proposed method, a series of experiments were carried out. The schematic of the experimental equipment is shown in Figure 7. It includes a rotating platform, an excitation coil, sensors, a signal conditioning circuit, a data acquisition card, a receiving terminal (a personal computer here) and electric machinery. Many defects are distributed on the edge surface of a rotating platform. A magnetizing yoke with an excitation coil is used to generate a magnetic field. We have found that vibration judgment error will rapidly decay with the increasing of the magnetic sensor's lift-off value. Therefore, setting a large lift-off value can effectively restrain the error caused by the random vibration of the detecting mechanism. Additionally, the noise judgment error will sharply increase with the increasing of the magnetic sensor's lift-off value. Thus, when setting a small lift-off value, the signal-to-noise ratio (SNR) of the detection signal is large and the sensitivity of MFL detection system is high. Here, comprehensively considering the influence of various kinds of error sources, the magnetic pole is a 1 mm distance away from the rotating platform. The Hall sensor probe is located at the centre of the two magnetic poles of the magnetizing yoke at a 0.5 mm distance away from the edge surface, aiming to acquire MFL signals. After being regulated by the signal conditioning circuit, MFL signals are transmitted to the data acquisition card. Finally, the computer receives them. In addition, the speed of the rotating platform is controlled by electric machinery.

The type of material of the top surface of the rotating platform is U71Mn (steel rail, 71-Manganese material). Defects with different sizes are distributed on the top surface of the rotating platform with its speed ranging from 2 to 50 m/s. The types of Hall effect sensors and data acquisition card are UGN3503 and ADLINK DAQ 2204. As the amplitude of the MFL signal is of a millivolt level, while the data acquisition card operates at the volt level, an AD620 instrumentation amplifier is applied to design an amplifying circuit, whose amplification factor is 100. In addition, to avoid the detection device magnetizing the rotating platform repeatedly, we lay out the magnetization reversal device opposite to the detection device.

**Figure 8.**
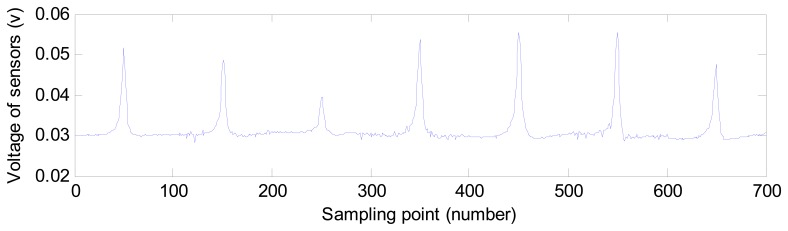
The experimental MFL signal gathered by sensors.

Figure 8 shows the experimental MFL signals gathered by sensors on groove defects. Different from simulated MFL signals, the experiment MFL signals include noise that appears when the Hall sensors acquire the signals.

**Figure 9.**
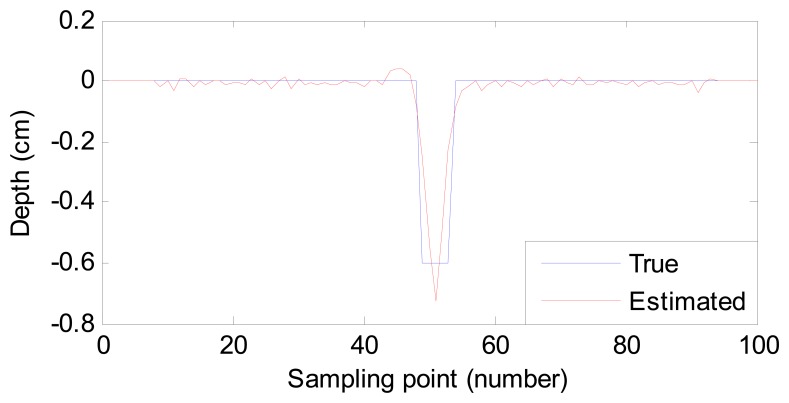
Comparison of the true profile and estimated profile (Sample 1).

**Figure 10.**
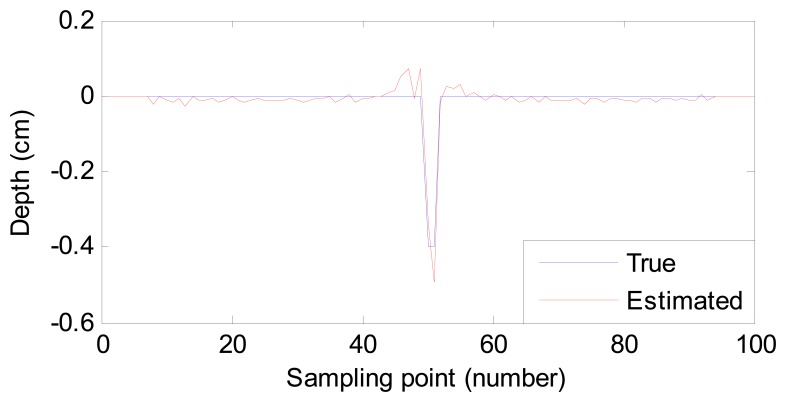
Comparison of the true profile and estimated profile (Sample 2).

Two estimated defect profiles for Sample 1 (0.04 cm width, 0.6 cm depth) and Sample 2 (0.02 cm width, 0.4 cm depth) are compared with the true ones by processing the experimental MFL signals shown in Figures 9 and 10. The RMSE values and estimation time are listed in Table 3. From them, we conclude that:

(1) Despite the existence of noise in the experimental MFL signals, the profiles estimated by the proposed method are close to the true ones. The result shows that the method based on MAPA is robust in the face of noise.
(2) The time to estimate defect profiles is as short as that for simulation data. The method is thus suitable for fast inspection in an industrial environment.
(3) The RMSE value decreases with the decrease of the defect size. The proposed method can achieve more accurate results for small-sized defect estimation.

## 5. Conclusions

In this paper, to estimate the defect profiles from MFL signals and meet the online inspection requirements, MAPA is proposed and applied to defect profile estimation. The major contribution of this paper is to achieve fast estimation of defect profiles from the magnetic flux leakage signal under the premise that the estimated profiles are clearly close to the desired ones in a noisy environment. The process of profile estimation includes the regulation of a weight vector in the proposed MAPA filter and the profile estimation with the filter.

Both simulation and experimental data are used to verify the effect of the proposed method. The results validate that the defect profile estimating method based on MAPA achieves high performance and robustness despite noise polluted signals. The presented method can deliver both fast estimation speed and reasonable prediction accuracy.

## Figures and Tables

**Table 1.** The values of parameters for multi-power APA (MAPA).

**Parameter**	**Value**
*N*_1_ and *N*_2_	4
*L*_1_ and *L*_2_	3
*μ*	0.1
*γ*	1 × 10^−5^
*P*	2

**Table 2.** RMSE values and the estimating time.

**Defect Sample**	**Defect Size**		**RMSE**	**Estimating Time (s)**

	**Width (cm)**	**Depth (cm)**		
1	2.54	0.90	0.0345	4.70 × 10^−4^
2	2.54	1.52	0.0426	4.63 × 10^−4^
3	3.56	0.51	0.0293	4.81 × 10^−4^
4	3.56	1.40	0.0472	4.67 × 10^−4^
5	4.57	0.38	0.0275	4.60 × 10^−4^
6	4.57	1.65	0.0467	4.67 × 10^−4^
7	5.59	1.52	0.0585	4.68 × 10^−4^
8	6.60	1.52	0.0674	4.62 × 10^−4^
9	7.62	1.65	0.0633	4.80 × 10^−4^
10	8.64	0.38	0.0417	4.72 × 10^−4^

**Table 3.** RMSE values and the estimating time from experimental signals.

**Defect Size**		**RMSE**	**Estimating Time (s)**

**Width (cm)**	**Depth (cm)**		
0.04	0.6	0.0522	4.13 × 10^−4^
0.02	0.4	0.0190	4.02 × 10^−4^
